# PPAR Gamma in Neuroblastoma: The Translational Perspectives of Hypoglycemic Drugs

**DOI:** 10.1155/2016/3038164

**Published:** 2016-10-05

**Authors:** Serena Vella, Pier Giulio Conaldi, Tullio Florio, Aldo Pagano

**Affiliations:** ^1^Department of Laboratory Medicine and Advanced Biotechnologies, IRCCS-ISMETT (Istituto Mediterraneo per i Trapianti e Terapie ad Alta Specializzazione), Palermo, Italy; ^2^Fondazione Ri.MED, Palermo, Italy; ^3^Section of Pharmacology, Department of Internal Medicine (DiMI) and Center of Excellence for Biomedical Research (CEBR), University of Genova, Genova, Italy; ^4^Department of Experimental Medicine (DIMES), University of Genova, Genova, Italy; ^5^IRCCS-AOU San Martino-IST, Genova, Italy

## Abstract

Neuroblastoma (NB) is the most common and aggressive pediatric cancer, characterized by a remarkable phenotypic diversity and high malignancy. The heterogeneous clinical behavior, ranging from spontaneous remission to fatal metastatic disease, is attributable to NB biology and genetics. Despite major advances in therapies, NB is still associated with a high morbidity and mortality. Thus, novel diagnostic, prognostic, and therapeutic approaches are required, mainly to improve treatment outcomes of high-risk NB patients. Among neuroepithelial cancers, NB is the most studied tumor as far as PPAR ligands are concerned. PPAR ligands are endowed with antitumoral effects, mainly acting on cancer stem cells, and constitute a possible add-on therapy to antiblastic drugs, in particular for NB with unfavourable prognosis. While discussing clinical background, this review will provide a synopsis of the major studies about PPAR expression in NB, focusing on the potential beneficial effects of hypoglycemic drugs, thiazolidinediones and metformin, to reduce the occurrence of relapses as well as tumor regrowth in NB patients.

## 1. Introduction 

*(1) Neuroblastoma.* Neuroblastoma (NB) is a tumor of the developing sympathetic nervous system observed in early childhood, which is characterized by a broad spectrum of clinical behaviors, ranging from complete regression to death.

NB represents the second most common extracranial malignancy of childhood, accounting for 8 to 10% of all childhood cancers (NB prevalence is about one case in 7,000–10,000 live births) and for approximately 15% of the pediatric deaths for malignant conditions [1].

The clinical presentation of NB ranges from asymptomatic masses to primary tumors that cause critical illness due to local invasion and/or widely disseminated disease. Most primary NB (65%) usually present in the abdominal region, often in the adrenal medulla. Other common sites of disease include the neck and head (5%), chest (20%), and pelvis (5%) [1].

NB is a disease of the sympaticoadrenal lineage of the neural crest and originates from neuroblasts in the developing peripheral nervous system [2].

In recent years, it has been suggested that NB tumorigenesis is dependent on the presence of cancer stem cells (CSCs), which have been also isolated from NB cell lines [3, 4]. CSCs are thought to be also responsible for metastasis and recurrence in NB patients [5, 6].

Cellular heterogeneity is a hallmark of NB nodules and the prognosis of these tumors depends on their differentiation levels [7]. Interestingly, cell lines established from several human NB retain similar cellular heterogeneity.

Biedler et al. described three cell subtypes, often discernible also in NB cell line cultures, based on cell morphology, biochemical features, and growth patterns [8]: (i) N-type (neuroblastic: aggregated, poorly attached, and rounded cells with short neurites); (ii) S-type (substrate-adherent and nonneuronal cells); and (iii) I-type (intermediate: mildly adherent cells, showing marked stem-like traits, representing cancer stem-like cells population, and being thought to originate both S- and N-type cells) [7].

Several studies have shown that these cell types derive from a common precursor and are able to bidirectionally differentiate. This bidirectional conversion between well-defined differentiation lineages of the neural crest has been termed “transdifferentiation” [9].

Because the transdifferentiation process is able to also allow the differentiating of malignant CSCs into benign phenotype, a novel concept in cancer biology was introduced: “induction of differentiation” as possible treatment (e.g., using retinoids to treat NB and acute promyelocytic leukaemia [10]).

The cause of NB development is still unclear occurring mostly as sporadic disease but also rare (about 1% of all cases) familial cases were reported [1]. Genomic alterations are associated with NB development and/or progression, many of which have proven to be correlated with clinical outcome. The most widely studied cytogenetic alterations, associated with poor outcome in NB, include N-myc oncogene amplification, loss or rearrangement of the distal portion of the short arm of chromosomes 1 (1p31-term), 3 (3p22), and 11 (11q23), gains of chromosome arm 1q or 17q, and the expression of the TrkB neurotrophin receptor and its ligand [34–38]. Other cytogenetic and molecular abnormalities are likely involved in NB pathogenesis and their identification could be useful for diagnosis, prognosis, and therapy of NB patients.

Traditional NB treatments include surgery, chemotherapy, radiotherapy, and biotherapy [34]. However, the majority of NB patients (50%) have poor outcomes and relapses, remaining a clinical challenge.

Unfortunately, in many cases, by the time of diagnosis, the disease has usually spread already. In these cases, the mainstay treatment is frequently intensive regimens including combinations of high doses of chemotherapeutics [39] that often are accompanied by unacceptable high toxicities and no long-term improvements. Innovative approaches are therefore needed for this disease. The new treatment protocols for NB currently under investigation consist of targeted radiotherapy and retinoid compounds (to induce terminal differentiation of NB cells), immunological treatment, such as using antidisialoganglioside 2 with or without association with cytokines (GM-CSF, IL-2), antiangiogenics, neurotrophin-signaling inhibitors, proapoptotic agents, allogeneic haemopoietic stem cell transplantation, and new chemotherapeutics [34, 39].

*(2) PPARs*. Peroxisome proliferator-activated receptors (PPARs) are ligand-activated transcription factors belonging to the nuclear receptor superfamily.

PPARs are activated by fatty acids, eicosanoids, other dietary lipids, and their metabolites, or synthetic ligands [40], which have been pharmacologically used in several diseases, making PPARs attractive therapeutic targets.

There are three PPAR isoforms (*α*, *β*/*δ*, and *γ*) which are encoded by separate genes [41] and are expressed during different stages of prenatal development [42].

Through the regulation of the expression of multiple genes [43], PPARs control several physiological processes, including cell proliferation, morphogenesis, differentiation, and cellular homeostasis [44, 45], and have been implicated in different human diseases such as hyperlipedimia, diabetes, obesity, inflammation, neurodegenerative disorder, cardiovascular diseases, and cancer [46–49].

Although all PPAR isoforms display a partially overlapping spectrum of activity, essentially as far as the control of lipid and energy metabolism is concerned, they differ in tissue expression pattern and functional roles [50–52].

PPAR-*α* is predominantly expressed in metabolically active tissues, such as liver, skeletal muscle, heart, intestinal mucosa, brown adipose tissue, adrenal gland, pancreas, and kidney. This receptor regulates catabolism of fatty acids and promotes lipolysis and fatty acid oxidation [53–56].

PPAR-*α* endogenous ligands (fatty acids and several fatty-acid-derived compounds) or synthetic pharmacological agonists (fibrate drugs, WY14643 and GW7647) have been identified [57], and some of them are currently used for the treatment of hypertriglyceridemia and cardiovascular diseases [58].

PPAR-*β*/*δ* is ubiquitously expressed particularly in liver, intestine, kidney, abdominal adipose tissue, skeletal muscle, and macrophages. It regulates energy expenditure, participating in fatty acid oxidation and regulating blood cholesterol concentrations and glucose levels [41, 54, 59].

PPAR-*β*/*δ* agonists are prostacyclin PGI2, oleic acid, and synthetic agents, such as GW501516, GW7842, and GW0742, which attenuate hepatic steatosis [60].

PPAR-*γ* is expressed within adipose tissue, the large intestine, spleen, skeletal muscle, liver, pancreas, endothelial cells, immune cells, various cancer cells, and brain [61–63].

It regulates energy storage and has a key role in fatty acid metabolism and glucose homeostasis [55, 64–66], mitochondrial biogenesis, and ROS metabolism [67, 68]. Many lipids, including eicosanoids and the cyclopentenone prostaglandin 15-deoxy-Δ12,14-prostaglandin J2 (15-deoxy-PGJ2), are endogenous PPAR-*γ* ligands, while synthetic agonists include thiazolidinediones (TZDs), GI262570, GW1929, and GW7845 [69]. PPAR-*γ* has been extensively studied as a pharmacological target in several diseases.

TZDs are the best-characterized pharmacological PPAR-*γ* agonists, and, among them, pioglitazone and rosiglitazone have been approved by FDA for treatment of type II diabetes [70–72].

In addition, another oral hypoglycemic drug, metformin, which directly improves insulin action, modulating AMPK activity (a key energy regulator), increases PPAR-*γ* mRNA levels [73], acting similarly to rosiglitazone [74].

## 2. PPARs in Neuroblastoma

Interestingly, it has been suggested to use PPARs as target for cancer treatment, and several PPAR agonists, in particular acting on PPAR-*γ*, represent promising therapeutic tools as antitumoral agents [75]; PPAR-*γ* agonists were reported to inhibit cell growth and to induce apoptosis in several cancer cell lines* in vitro* and* in vivo* [76–86], including NB cells [87, 88].

All three PPARs isoforms have been identified in NB, although human NB cell lines express PPAR-*α* (mRNA or proteins) at very low level [11, 89–91], and PPAR-*β*/*δ* expression data are still incomplete [11, 92]. Conversely, PPAR-*γ* is highly expressed in NB cell lines [11, 12] and in primary NB cell cultures [75], being mainly localized in the nuclei rather than in the cytoplasm and being particularly expressed in cells showing ganglionic differentiation [11, 89].

In addition, it has been documented that embryonic rat brain and neural stem cells have higher concentration of PPAR-*γ* than adult rat brain [42, 93]. In neural stem cells, PPAR-*γ* is involved in the regulation of proliferation and differentiation [94].

Interestingly, PPAR-*γ* expression is correlated to the maturational stage of NB and therefore to NB patients' outcome [85], and PPAR-*γ* agonists induce NB cell differentiation, inhibiting proliferation, neurite outgrowth, and reducing N-myc levels [11].

### 2.1. PPARs Agonists in Neuroblastoma

Several studies have assessed the activity of PPAR-*γ* in NB, evaluating the effects of several natural or synthetic ligands on cellular proliferation, apoptosis, and differentiation (Table 1).

15-deoxy-Δ12,14-prostaglandin J2 (15-deoxy-PGJ2), a high-affinity natural ligand of PPAR-*γ*, inhibits* in vitro *growth and induces apoptosis in NB cells [11–16], through PPAR-*γ*-dependent ERK2 activation, although PPAR-*γ*-independent effects of 15-deoxy-PGJ2 have been also described [14].

In addition, Rodway et al. have found that the inhibition of NB growth induced by 15-deoxy-PGJ2 can be reduced by the presence of serum lysolipids in the culture medium [14], while Emmans et al. reported that the degree of PPAR activation, due to 15-deoxy-PGJ2, in a NB cell line, is attenuated in the presence of the retinoblastoma protein (Rb) and restored by treatment with the histone deacetylase inhibitor trichostatin A (TSA). The combination treatment with 15-deoxy-PGJ2 and TSA enhances the inhibition of NB growth, suggesting a synergistic activity of the two compounds [13]. Furthermore, 15-deoxy-PGJ2 promotes NB cell differentiation, which may be mediated by the p38 MAP kinase activation and the AP-1 signaling pathway [95].

Synthetic PPAR-*γ* ligands have been also tested to contrast NB cell growth.

Han and coworkers firstly evaluated the effect of the synthetic ligand, GW1929, in the NB cell line LA-N-5, and found that this compound induces cell differentiation and inhibits proliferation [11].

GW1929 prodifferentiating effect was shown to be dependent on PPAR-*γ* activation, as demonstrated by the use of specific antagonists [96].

In 2005, Valentiner et al. tested the effects of four TZDs (ciglitazone, pioglitazone, troglitazone, and rosiglitazone) in seven NB cell lines (i.e., Kelly, LAN-1, LAN-5, LS, IMR-32, SK-N-SH, and SH-SY5Y) [17]. All the ligands, in particular ciglitazone and rosiglitazone, inhibited cell proliferation and viability in a dose-dependent manner, with different drug effectiveness among cell lines. Moreover, drug potency was not related to PPAR-*γ* protein amount in NB cell lines, but rather to various cellular conditions associated with the receptor function.

The antiproliferative effect of rosiglitazone was confirmed by the same group* in vivo,* in a metastatic xenograft mouse model [18], although its antitumor effect was very limited.

Ciglitazone was also used in association with 15-deoxy-PGJ2 to overexpress Rb protein and inhibit PPAR-*γ* activity, reducing NB cell growth [13].

Servidei et al. tested 15-deoxy-PGJ2 and rosiglitazone on 8 NB cell lines, with different phenotypes, including N- and S-types [12]. The two PPAR-*γ* ligands inhibit cell growth in all cell lines, and the sensitivity seems to be more associated with the cell phenotype than with PPAR-*γ* expression: indeed, N-type cells are more susceptible to treatment than S-type cells, partly because of their higher capability of undergoing apoptosis.

Many studies have documented that the inhibitory effects of TZDs on neuroblastoma cell growth are partially due to an increase of apoptosis. Indeed, troglitazone induced PPAR-*γ*-dependent apoptosis in NB-1 cells [19] and in SHEP NB [20]. In contrast, only two studies, to our knowledge, reported antiapoptotic effects of rosiglitazone which protected NB cells subjected to MPP^+^-induced mitochondrial injury reducing ROS production [21, 22].

Proapoptotic effects of rosiglitazone were also reported [23]. This PPAR-*γ* ligand significantly inhibits cell adhesion and invasiveness and induces apoptosis, more effectively in SK-N-AS than in SH-SY5Y cell lines. The distinct response of the two NB cell lines is likely due to a reduced phosphorylation of PPAR-*γ* and consequently its increased activity in SK-N-AS cells. Cellai and colleagues also evaluated the* in vivo* effect of TZDs in NB xenograft models, confirming their previous* in vitro* observations [24]. Indeed rosiglitazone (150 mg/kg/day) for 4 weeks significantly reduced tumor growth (−70%) as compared to control mice [24].

In addition, rosiglitazone induces differentiation, increasing density of dendritic spines in rat primary cortical neurons [25].

Moreover, in neural stem cells (NSC) from adult mammalian brain, pioglitazone and rosiglitazone directly regulate proliferation, differentiation, and migration [26].

Accordingly, Miglio et al. described the effects of pioglitazone on SH-SY5Y NB cells, in which this agonist promotes differentiation and outgrowth of cell processes, in a dose-dependent manner [27]. In 2014, Chiang et al. evaluated the effects of rosiglitazone in the mouse NB Neuro 2a (N2A) cell line. This agonist stimulates neurite outgrowth and significantly increases the population of neurite-bearing cells, via PPAR-*γ* pathway [28].

All these observations are in agreement with previous findings indicating that PPAR-*γ* activation contributes to neuronal differentiation [11, 94–99].

While PPAR-*γ* activation mainly results in apoptosis promotion in proliferating cells, PPAR-*β*/*δ* natural (i.e., oleic acid) and synthetic (i.e., GW0742) agonists induce G1 cell cycle arrest, reduce cell migration and invasiveness, and increase neuronal differentiation in SH-NH-5YSY [29].

In summary, all these results suggest the possible use of PPAR agonists as novel therapy for NB, but to date clinical trials are not yet underway (http://www.who.int/topics/clinical_trials/en/).

### 2.2. Metformin and Neuroblastoma

Beyond TZDs, metformin is another hypoglycemic drug able to modulate PPAR expression or activity, although these effects are rather cell specific and mainly indirectly mediated by the activation of AMPK.

Metformin is biguanide with a well-known safety profile, mainly used as oral antidiabetic drug [100, 101], whose promising anticancer activity was recently discovered [102]. It is well-documented that metformin inhibits tumor growth in* in vitro* and* in vivo* preclinical cancer models [103–110], and various human clinical trials are in progress (WHO International Clinical Trials Registry Platform, http://www.who.int/topics/clinical_trials/en/).

In particular, metformin seems to selectively affect cancer stem cell survival, inhibiting cancer metastases and thus represents a good potential adjuvant agent for chemotherapy (as reviewed by [111]).

However, the molecular mechanisms of action of metformin are still not completely defined, although it seems that the antiproliferative mechanisms induced by this drug are at least partially diverging from those regulating glucose homeostasis. While the latter is mainly dependent on the AMPK activation, the antitumor activity of metformin is mediated by inhibition of AKT/mTOR (also involving AMPK), the inhibition of TK activity, or the regulation of chloride channels [112–116].

As far as the effects of metformin on PPAR activity are concerned, several studies were performed but the results are extremely dependent on the receptor subtype and the cells analyzed. For example, metformin increased PPAR-*β*/*δ* expression in muscle cells and activity in endothelial cells [117, 118], reducing the effects of ER stress and increasing the bioavailability of nitric oxide [119]. On the other hand, metformin counteracts antiosteogenic PPAR-*γ* activation by rosiglitazone in bone marrow progenitor cells [120] and both PPAR-*γ* and PPAR-*α* activity in hepatoma cells [121]. Finally, a PPAR-*α* activation role of metformin was identified to increase GLP-1 receptor levels [122].

However, although demonstrated in several models, the role of the modulation of PPAR expression and/or activity in the antiproliferative effects of metformin in neuroblastoma has not been addressed yet.

The effect of metformin on NB was firstly demonstrated by our groups [30].

We reported that the effects of metformin treatment in human SKNBE2 and SH-SY5Y NB cell lines are a significant reduction in the proliferation rate and cell viability, due to inhibition of AKT phosphorylation and an increased cell death, via apoptosis-independent pathways. These effects were more pronounced in SKNBE2, which are less differentiated, highly proliferative cells than SH-SY5Y cells. Notably, metformin effects were different depending on the differentiating stimuli, being abolished by retinoic acid, but were potentiated by overexpression of NDM29, a noncoding RNA affecting NB malignancy, although both conditions were characterized by a neuron-like differentiated phenotype [30, 123–128].

These* in vitro* results are in agreement with those of Kumar and coworkers who evaluated the antitumor activity of metformin against neuroblastoma* in vivo* [31]. Oral administration of metformin, in both SH-SY5Y and SK-N-BE xenograft NB mouse models, significantly inhibited the growth of the tumors. NB cell viability is reduced by metformin, which also interferes with spheroid formation in 3D cultures, confirming that its antitumor effect could also involve the inhibition of CSC self-renewal. Moreover, in this study, it was confirmed that SKNBE2 cells are more susceptible to metformin than SH-SY5Y cells. In addition, studying the underlying signaling mechanisms, they highlighted that a modulation of Rho-GTPases and MAP kinase activation mediate metformin effects on NB cell survival.

In the same year, Vujic et al. successfully used metformin to inhibit cell proliferation and induce apoptosis in NRAS mutant NB cell lines (SK-N-AS and CHP-212), in which NRAS signaling is constitutively active through the PI3K/AKT/mTOR pathway [32].

In 2015, Mouhieddine and colleagues found that metformin reduces proliferation rate, viability, and invasive potential of NB cell lines SH-SY5Y [33].

Interestingly, focusing on metformin effect on stem cell population within a 3D culture model, these authors reported that this drug is able to decrease, but not abolish, cell sphere-forming ability, significantly targeting and reducing cancer stem/progenitor cell population and thus possibly preventing recurrence.

Notably, metformin also reduces MMP-2, a protein involved in extracellular matrix degradation, favoring metastasis and cancer progression [33].

Thanks to the highly selective and cytotoxic effects of metformin on NB cells and on stem cell population in particular (Table 2), these studies suggest that high-risk NB patients could have great benefit by using metformin in terms of increasing disease prognosis and propose that this drug can also be used as a novel therapeutic agent against neuroblastoma. However, at the moment, there are no clinical trials evaluating metformin efficacy for the treatment of NB (WHO International Clinical Trials Registry Platform, http://www.who.int/topics/clinical_trials/en/).

## 3. Conclusion 

Despite advances in standard therapeutic protocols, the prognosis of NB has not gained significant progress, especially concerning the rates of metastasis, the incidence of recurrences, and the long-term survival, which are all correlated with the presence of CSCs.

Recently, many groups focused their attention on PPARs, suggesting the use of several PPAR agonists, currently used as hypoglycemic drugs, for NB treatment. Interestingly, many* in vitro* and* in vivo* studies reported the successful use of PPAR agonists, and in particular of TZDs, in the inhibition of NB cell proliferation and tumor growth, in the induction of cell death, and in the promotion of cell differentiation. These effects are PPAR-dependent, even if some PPAR-independent effects have been also described. Notably, PPAR agonists also reduce the viability of CSCs, the tumor cell subpopulation responsible of NB recurrence. Similar results were obtained with another hypoglycemic drug, metformin, which acts also via the modulation PPAR activity. In the future, it will be important to examine the contribution of PPARs in the antiproliferative activity of metformin and the possible synergistic effect with known or novel selective PPAR agonists.

Although further studies* in vitro* as well as* in vivo* are needed, these hypoglycemic drugs may represent realistic new therapeutic approaches to be used as good adjuvant chemotherapeutic agents in NB treatment.

## Figures and Tables

**Table 1:** Preclinical and experimental studies on PPAR agonists in neuroblastoma.

Drug/s	Reference/s	Year	Target	Study types	Cell lines/animal model	Effects
15-deoxy-PGJ2	[11–16]	2001, 2003, 2004	PPAR-*γ*	*In vitro*	NB cell lines and primary cultures of cortical neurons	Inhibition of growth and apoptosis induction, through PPAR-*γ*-dependent and PPAR-*γ*-independent effects.

GW1929	[11]	2001	PPAR-*γ*	*In vitro*	LA-N-5	Prodifferentiating effect and inhibition of proliferation.

Rosiglitazone and 15-deoxy-PGJ2	[12]	2004	PPAR-*γ*	*In vitro*	SH-SY5Y, SH-EP1, SK-N-AS, SK-N-FI, LA-N-5, SMS-KCNR, SK-N-DZ, and LA-N-1	Inhibition of cell growth with different sensitivity related to the cell phenotype.

Ciglitazone and 15-deoxy-PGJ2	[13]	2004	PPAR-*γ*	*In vitro*	SK-N-AS, IMR-32, SK-N-SH, and ND-7	Overexpression of Rb protein and inhibition of PPAR-*γ* activity, reducing NB cell growth.

Ciglitazone, pioglitazone, troglitazone, and rosiglitazone	[17]	2005	PPAR-*γ*	*In vitro*	Kelly, LA-N-1, LA-N-5, LS, IMR-32, SK-N-SH, and SH-SY5Y	Inhibition of cell proliferation and viability in a dose-dependent manner.

Rosiglitazone	[18]	2010	PPAR-*γ*	*In vivo*	SK-N-SH xenograft NB mouse model	Inhibition of tumor growth.

Troglitazone	[19]	2002	PPAR-*γ*	*In vitro*	NB-1 cell line	Increase of PPAR-*γ*-dependent apoptosis.

Troglitazone	[20]	2006	PPAR-*γ*	*In vitro*	SHEP NB cell line	Increase of PPAR-*γ*-dependent apoptosis.

Rosiglitazone	[21, 22]	2006, 2007	PPAR-*γ*	*In vitro*	SH-SY5Y cell line	Antiapoptotic effects of rosiglitazone which protected NB cells subjected to MPP^+^-induced mitochondrial injury reducing ROS production.

Rosiglitazone	[23]	2006	PPAR-*γ*	*In vitro*	SK-N-AS and SH-SY5Y cell lines	Inhibition of cell adhesion, invasiveness, and proapoptotic effects.

Rosiglitazone	[24]	2010	PPAR-*γ*	*In vivo*	SK-N-AS xenograft NB mouse model	Significant decrease of tumor growth (−70%) as compared to control mice.

Rosiglitazone	[25]	2008	PPAR-*γ*	*In vitro*	Rat primary cortical neurons	Induction of cell differentiation, increasing dendritic spine density.

Pioglitazone and rosiglitazone	[26]	2011	PPAR-*γ*	Both *in vitro* and *in vivo*	Adult male Wistar rats	Induction of proliferation, differentiation, and migration of neural stem cells *in vitro* and *in vivo.*

Pioglitazone	[27]	2009	PPAR-*γ*	*In vitro*	SH-SY5Y cell line	Induction of differentiation and neurite outgrowth, promoting differentiation and outgrowth of cell processes.

Rosiglitazone	[28]	2014	PPAR-*γ*	*In vitro*	Mouse NB Neuro 2a (N2A) cell line	Stimulation of neurite outgrowth and significant increase of the population of neurite-bearing cells, via PPAR-*γ* pathway.

Oleic acid or GW0742	[29]	2007	PPAR-*β*/*δ*	*In vitro*	SH-NH-5YSY cell line	Induction of G1 cell cycle arrest, reduction of cell migration and invasiveness, and an increase of neuronal differentiation.

**Table 2:** Studies on metformin treatment to date in neuroblastoma.

Reference/s	Year	Study types	Cell lines/animal model	Effects
[30]	2014	*In vitro*	SKNBE2 and SH-SY5Y cell lines	Significant reduction in the proliferation rate and cell viability, due to inhibition of AKT phosphorylation, and an increased cell death, via apoptosis-independent pathways.

[31]	2014	*In vivo*	SH-SY5Y and SK-N-BE xenograft NB mouse models	Significant inhibition of tumor growth and NB cell viability, interfering with spheroid formation in 3D cultures. Modulation of Rho-GTPases and AMPK activation mediate metformin effects on NB cell survival.

[32]	2015	*In vitro*	SK-N-AS and CHP-212 cell lines	Inhibition of cell proliferation and induction of apoptosis.

[33]	2015	*In vitro*	SH-SY5Y cell line	Reduction of proliferation rate, viability, and invasive potential.
